# The Efficacy of *Ganoderma lucidum* Extracts on Treating Endometrial Cancer: A Network Pharmacology Approach

**DOI:** 10.1007/s43032-024-01500-3

**Published:** 2024-03-06

**Authors:** Min Shi

**Affiliations:** Department of Medical Oncology, Zhejiang Putuo Hospital, Zhoushan, 316100 Zhejiang Province China

**Keywords:** *Ganoderma lucidum* extracts, Endometrial cancer, Network pharmacology, GO and KEGG enrichment, Rap1 signaling pathway

## Abstract

*Ganoderma lucidum* (GL) is a prominent medicinal mushroom in traditional Chinese medicine, known for its potent antitumor properties. This study aimed to illustrate the efficacy of GL extracts (GLE) on treating endometrial cancer (EC) and explore the underlying mechanisms via network pharmacology and experimental validation. Network pharmacological analysis was conducted to explore the therapeutic efficacy and mechanisms of GL on EC. In vitro experimental validation was performed on human endometrial cancer cell lines HEC-1-A and KLE. Network pharmacology revealed that key targets of GL against EC were primarily associated with the Rap1 signaling pathway. In in vitro experiments, GLE or GGTI-298 (a GTPase inhibitor) treatment inhibited cell proliferation and migration, promoted cell apoptosis, increased caspase-3 level, and arrested cell cycle in G1 phase in HEC-1-A and KLE cells. GLE increased the protein expression of Rap1-GTP, p-AKT, and p-ERK2 in HEC-1-A and KLE cells. Moreover, GGTI-298 enhanced the effects of GLE on suppressing the malignant progression of EC cells and on activating Rap1 signaling pathway. GLE inhibited the malignant progression of EC cells probably via activating the Rap1 signaling pathway.

**Supplementary Information:**

The online version contains supplementary material available at 10.1007/s43032-024-01500-3.

## Introduction

Endometrial cancer (EC) ranks as the fifth most prevalent gynecological malignancy, constituting for approximately 7% of all female cancers [1]. The global incidence of EC demonstrates an increasing trend, with nearly 417,000 new cases and 97,000 fatalities reported in 2020 [2]. Currently, surgery, chemotherapy, and endocrine therapy are the common strategies for EC treatment [3]. However, the therapeutic effects are still unsatisfactory probably due to drug resistance, metastasis, and recurrence. Hence, the identification of effective pharmaceutical agents and therapeutic targets is imperative for EC.

*Ganoderma*, also known as Lingzhi, is a highly esteemed herb in traditional Chinese medicine (TCM) and has been widely employed as a functional food and therapeutic agent [4]. *Ganoderma lucidum* (GL), a prominent species within the *Ganoderma* genus, exhibits a diverse array of pharmacological properties, including antiviral, anti-inflammatory, and antioxidative activities [5, 6]. Extensive research has revealed that GL displays remarkable antitumor effects against various types of cancers. Multiple studies have provided evidence supporting its efficacy in inhibiting tumor growth and proliferation [7–10]. Jin et al. [9] indicated that GL polysaccharide is a favorable therapeutic agent for cervical cancer via alleviating cell invasion and inducing apoptosis. A previous study demonstrated that a mixture of GL and *Agaricus Blazi Murill* exerts a repressive effect on the viability and proliferation of EC cells [11]. However, the effects and molecular mechanisms of GL extract (GLE) on EC are still needed to be deciphered.

Ras-associated protein-1 (Rap1) is a member of the Ras superfamily, which is a group of small GTPases. The activation of Rap1 and its downstream effectors has been associated with tumor progression, invasion, and the formation of metastatic lesions [12–17]. Understanding the mechanisms by which the Rap1 signaling pathway influences these processes holds promise for developing targeted therapeutic strategies against cancer. For example, in breast cancer, upregulation of Rap1 has been demonstrated to promote cell invasion and migration. Increased Rap1 activity promotes the rearrangement of the cytoskeleton and formation of cellular protrusions, facilitating the movement of cancer cells. This effect is thought to contribute to the metastatic potential of breast cancer cells by enabling their invasion into surrounding tissues and dissemination to distant sites [12]. Rap1 depletion can impede the signaling pathways involved in cell cycle progression and proliferation, thereby inhibiting the growth of non-small cell lung cancer cells [14]. Overexpression of Rap1GAP, a Rap1 activating protein, impairs cell viability and migration in gastric cancer, pancreatic cancer, and thyroid cancer [15–17]. Until now, the Rap1 signaling pathway has been revealed as a regulatory target of diverse anticancer drugs, such as *Cremastra appendiculata*, ivosidenib, and asparanin A [18–20]. However, whether GLE can protect against EC via regulating the Rap1 signaling pathway is still unclear.

Network pharmacology is an effective approach to elucidate the potential functions and mechanisms of TCM in treating diverse diseases by constructing compound-target-pathway networks [21]. The study utilized network pharmacology to identify the hub targets and bioactive compounds of GL against EC. Enrichment analysis was then applied to predict the corresponding mechanisms associated with GL treating EC. Following this, we investigated the anticancer effects of GLE on the malignant progression in EC cells. Moreover, we verified the contribution of the Rap1 signaling pathway to the underlying mechanism of GLE against EC. Our findings may highlight a promising therapeutic drug for EC and lay the groundwork for further investigation into its underlying mechanisms.

## Methods

### Screening Active Compounds and Corresponding Targets of GL

To screen active compounds and their corresponding targets, chemicals from GL were collected from the Traditional Chinese Medicine Information Database (TCM-ID, http://bidd.group/TCMID/) [22], the Traditional Chinese Medicine Database and Analysis Platform (TCMSP, https://tcmsp-e.com/) [23], and the Herb Ingredients’ Targets (HIT, http://lifecenter.sgst.cn/hit/) [24]. Final bioactive compounds were selected by evaluating their pharmacological properties, including Absorption, Distribution, Metabolism, and Excretion (ADME), and calculating their quantitative estimate of drug-likeness (QED) value [25–27]. The Food and Drug Administration in the DrugBank set the QED threshold at 0.2 [28]. Furthermore, corresponding targets of bioactive compounds were searched from TCM-ID, TCMSP, HIT, and the Search Tool for Interactions of Chemical (STITCH) databases. Target information obtained was then normalized to the National Center for Biotechnology Information (NCBI) database.

### Screening Targets Related to EC

EC-related targets were sourced from GeneCards (https://www.genecards.org/), DisGeNET (https://disgenet.org/), and the Online Mendelian Inheritance in Man (OMIM, https://www.omim.org/) databases.

### Interaction Network Construction

The intersection targets related to both GL and EC were obtained by establishing a Venn diagram. To create the compound-target network, we imported the data of bioactive compounds and their intersecting targets into Cytoscape 3.9.0 software. Moreover, the protein–protein interaction (PPI) network of intersecting targets was constructed using the STRING database (https://cn.string-db.org/) and Cytoscape. The top 10 hub targets were obtained using the CytoHubba algorithm according to the degree values of PPI network in Cytoscape 3.9.0.

### Gene Ontology (GO) and Kyoto Encyclopedia of Genes and Genomes (KEGG) Enrichment Analyses

The intersection targets of GL and EC were subjected to enrichment analysis using the GO and KEGG databases. A hypergeometric distribution model was utilized to estimate the significant correlation between the target gene set and the GO term/KEGG pathway [29, 30]. The *P* values, adjusted by Bonferroni correction, were used to assess the strength of association between the identified targets and the enriched GO terms/KEGG pathways. A *P* value less than 0.01 was considered statistically significant. The top 15 GO terms and KEGG pathways were visualized using bar plot diagrams. A target-biological process (BP)-pathway network, comprising intersecting targets, the top 15 GO-BP terms, and the top 15 ranked KEGG pathways, was constructed using Cytoscape 3.9.0.

### GLE Preparation

The GL mushrooms underwent freeze-drying and ground into a fine powder. For extraction, 20 g GL powder was mixed with 400 mL of ethanol and then shook at 150 rpm for 24 h using a rotating shaker (Aohua, China). The extracting solution was filtered using filter paper and the ethanol was evaporated. The obtained GLE was re-dissolved in ethanol to 200 mg/mL for subsequent cell experiments.

### Cell Culture and Treatment

Human EC cell lines (KLE and HEC-1-A) were obtained from the American Type Culture Collection (ATCC, VA, USA). Both HEC-1-A and KLE cells were cultured in Dulbecco’s Modified Eagle Medium/Nutrient Mixture F-12 (DMEM/F-12, Gibco, NY, USA) supplemented with 10% fetal bovine serum (FBS, Gibco, NY, USA). The cells were maintained in an incubator with 5% CO_2_ at 37 °C. The cells were subjected to different treatments, including ethanol, 0.1 mg/mL GLE, 0.5 mg/mL GLE, 1.0 mg/mL GLE, 15 μM geranylgeranyltransferase I inhibitor (GGTI-298), and a combination of 1.0 mg/mL GLE and 15 μM GGTI-298.

### Cell Counting Kit-8 (CCK-8) Assay

Cell proliferation was assessed using the Cell Counting Kit-8 (CCK-8) assay (Dojindo, Kumamoto, Japan). HEC-1-A and KLE cells (1 × 10^5^ cells/mL) were seeded into a 96-well plate (100 μL/well). After incubation for 12, 24, 36, and 48 h, the cells were incubated with 10 μL of CCK-8 reagent for an additional 2 h at 37 °C. The absorbance of cells was measured using a DR-200Bs microplate reader (Diatek, China) at 450 nm.

### Transwell Assay

Transwell migration assays were performed using the following procedure: HEC-1-A or KLE cells (2 × 10^4^ cells) were seeded into the upper chambers of transwell inserts. In the lower chambers, 600 μL of Dulbecco’s Modified Eagle Medium/Nutrient Mixture F-12 (DMEM/F-12) supplemented with 10% FBS was added. The cells were then incubated for 24 h. After incubation, the cells on the lower chambers were fixed with methanol at 4 °C for 30 min and stained with crystal violet for 20 min. The migrated cells were visualized and imaged using a light microscope (Olympus, Japan) in nine random fields.

### Flow Cytometry

The apoptosis and cell cycle were evaluated using flow cytometry as previously described [31]. For the apoptosis assay, 3 × 10^5^ cells were stained with Annexin V-FITC for 15 min propidium iodide (PI) for 10 min. To assess the cell cycle, cells were fixed with prechilled 70% methanol for 4 h at 4 °C and then incubated with 50 µg/mL PI at 37 °C for 30 min in the dark. The apoptosis and cell cycle were detected using a flow cytometer equipped with Cell Quest software (BD Biosciences, NJ, USA).

### Enzyme-Linked Immunosorbent Assay (ELISA)

The level of apoptosis-related protein caspase-3 in cells was determined using a caspase-3 ELISA kit (mlbio, China).

### Western Blotting

Western blotting was performed on cells according to the previously reported method [31]. Primary antibodies used for western blotting were anti-AKT, anti-phospho-AKT (p-AKT), anti-Rap1, anti-Rap1-GTP, anti-ERK2, anti-p-ERK2, and anti-GAPDH (1:1000, Abcam, UK). GAPDH served as an internal control.

### Statistical Analysis

The data were presented as mean ± standard deviation. Statistical analysis was performed using GraphPad Prism 7.0, including a one-way analysis of variance (ANOVA) to compare the groups, followed by Tukey’s test to determine significance at* P* < 0.05.

## Results

### GLE Inhibits the Proliferation of EC Cells

**Fig. 1:**
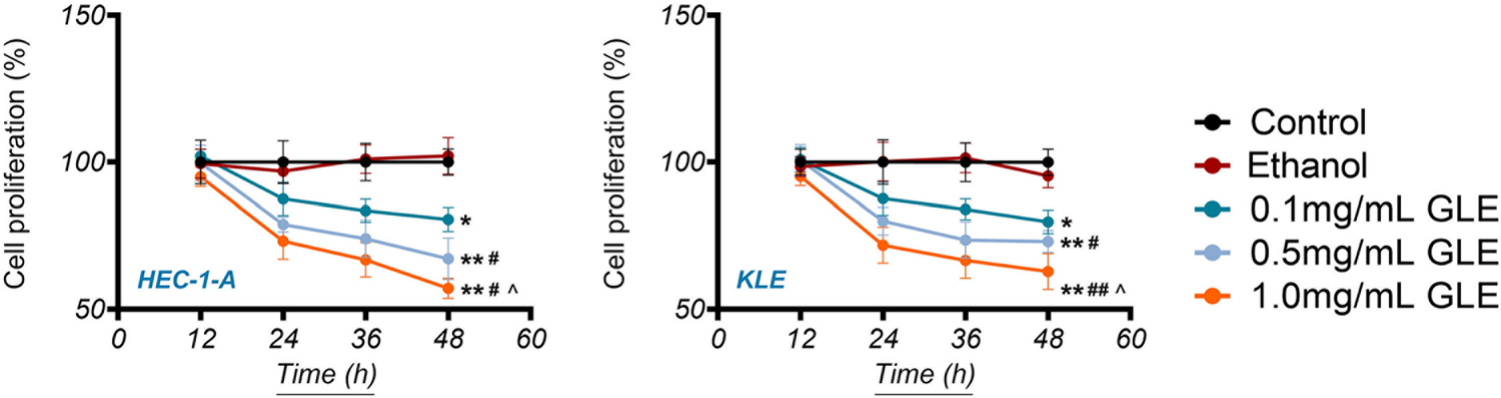
GL extracts (GLE) inhibit EC cell proliferation in a dose-dependent manner. The human EC cell lines (HEC-1-A and KLE) were treated with different dose of GLE (0.1, 0.5, and 1.0 mg/mL) for 12, 24, 36, and 48 h, and the cell proliferation was measured by CCK-8 assay. Data are presented as the mean ± standard deviation (SD) of three independent experiments. ^*^P < 0.05 and ^**^P < 0.01 *vs.* the control, ^#^*P* < 0.05 and ^##^*P* < 0.01 *vs.* the 0.1 mg/mL GLE treatment, and ^^^*P* < 0.05 *vs.* the 0.5 mg/mL GLE treatment

In order to determine the suppressive effect of GLE on EC development, we conducted an evaluation of the proliferation ability of HEC-1-A and KLE EC cell lines when treated with various concentrations of GLE (ranging from 0.1 to 1.0 mg/mL). Figure 1 reveals that the growth of both the HEC-1-A and KLE cells was suppressed in a dose-dependent manner following the administration of GLE, as compared to the control group (*P* < 0.05). Ethanol, the solvent used, had no impact on the proliferation of HEC-1-A and KLE cells. Among the various concentrations tested, 1.0 mg/mL GLE exhibited relatively superior efficacy in inhibiting the proliferation of EC cells (*P* < 0.05, Fig. 1). Consequently, the concentration of 1.0 mg/mL GLE was employed to administer treatment to both HEC-1-A and KLE cells in all subsequent experiments.

### Bioactive Compounds and Related Targets in GLE

A total of 75 compounds were obtained in GLE, as per the information gleaned from the publicly available databases TCM-ID, TCMSP, and HIT. These compounds were identified via ADME screening, and 26 out of the 75 compounds satisfied the suggested criteria QED > 0.2. The final selected 26 bioactive compounds were listed in Table 1 and included for further study. Additionally, a total of 1078 targets related to 26 bioactive compounds were obtained from public databases (TCM-ID, TCMSP, HIT, and STITCH).

**Table 1:** Thirty-five bioactive compounds of GL

Chemical	QED	Chemical	QED
Anethole	0.6262	Pentadecylic acid	0.4059
Caprylic acid	0.5818	Gamma-aminobutyric acid	0.3980
Nonanoic acid	0.5775	WLN: VH6	0.3957
Hexanoic acid	0.5687	Nonanal	0.3938
l-Carvone	0.5247	Lauric acid	0.3925
Ergosterol	0.5106	Decanal	0.3868
FUM	0.4992	Citral	0.3433
Ganoderic acid DM	0.4765	Stearic acid	0.3017
Nonane	0.4626	GCS	0.2966
Myristic acid	0.4490	EIC	0.2944
Beta-sitosterol	0.4354	MTL	0.2704
Ganoderic acid Df	0.4348	*trans*-2,4-Decadienal	0.2470
Hyacinthin	0.4290	Ganoderic acid T	0.2340

GL *Ganoderma lucidum*, QED quantitative estimate of drug-likeness

### EC-Related Targets

The targets associated with EC were identified via a comprehensive search of the GeneCards, DisGeNET, and OMIM databases. The search resulted in a total of 237 genes in GeneCards, 402 genes in DisGeNET, and 197 genes in OMIM. A total of 679 EC-related targets were obtained by removing duplicates.

### Identification of Potential Targets for GLE Against EC

**Fig. 2:**
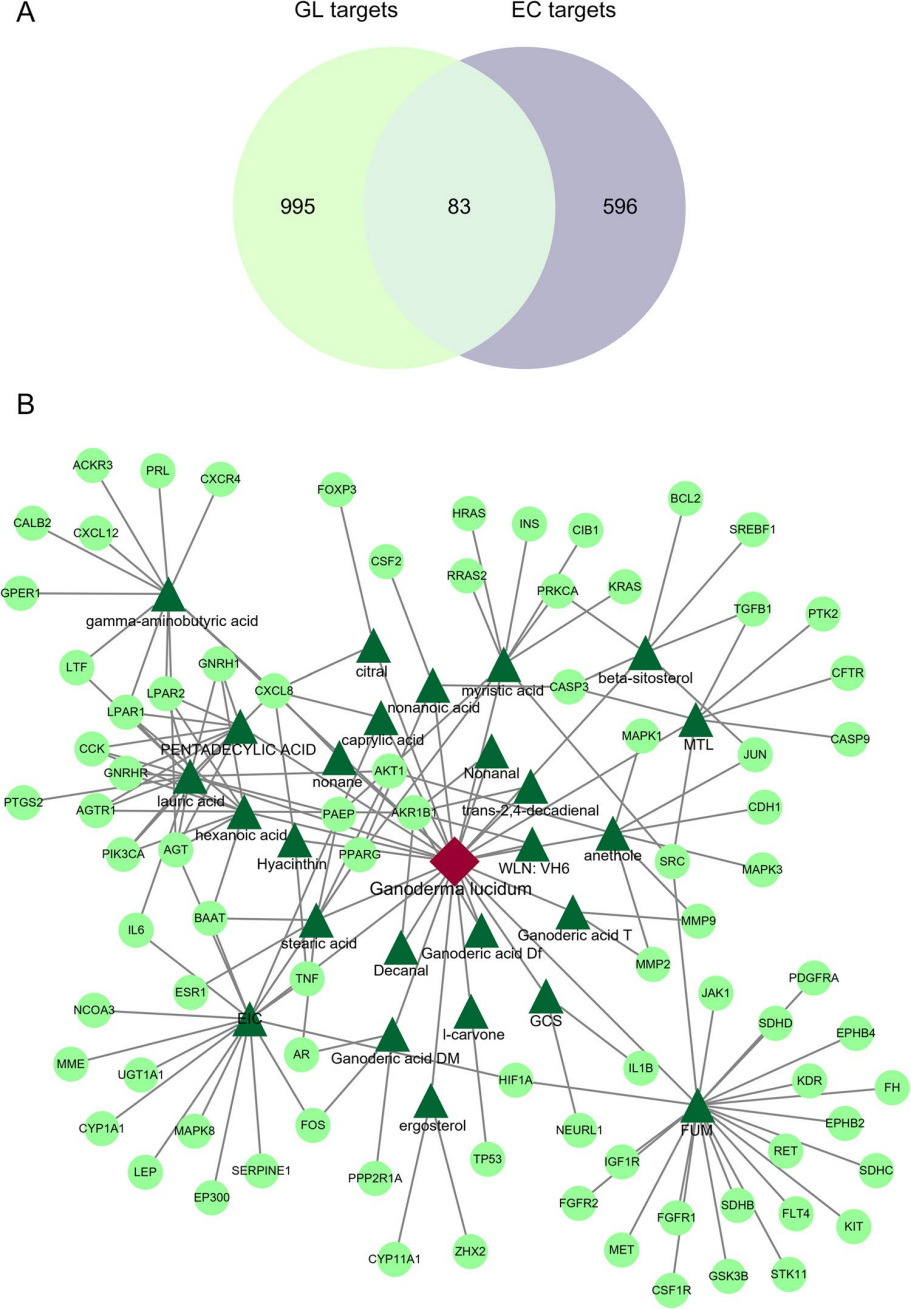
Compound-target networks of *Ganoderma lucidum* (GL) against endometrial cancer (EC). **A** Venn diagram of GL and EC-related targets. **B** Bioactive compound-target network of GL in treating EC

**Fig. 3:**
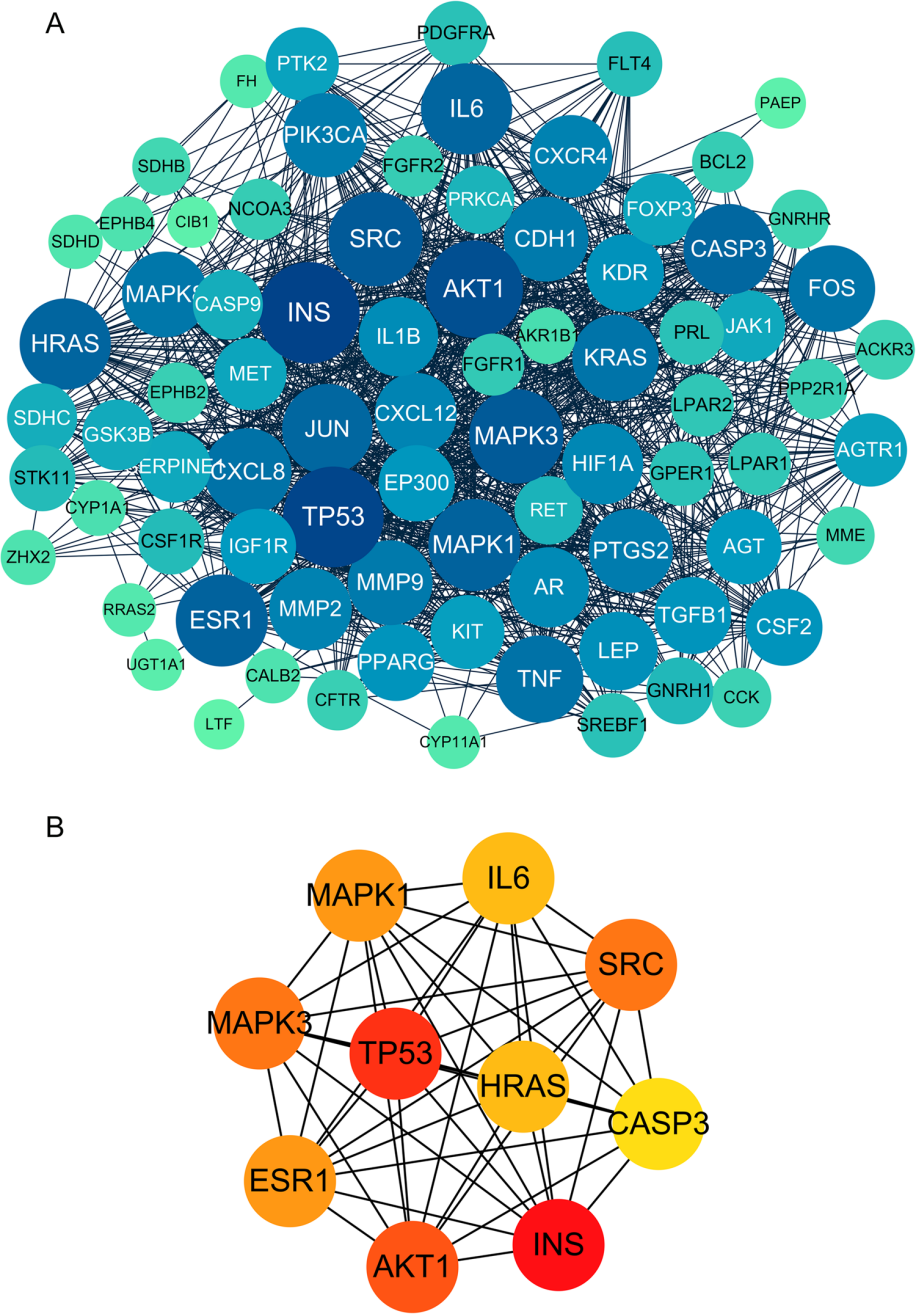
Protein–protein interaction (PPI) network of potential targets for GL against EC. **A** PPI network of 83 potential targets for GL in treating EC. **B** PPI network of top 10 hub targets

A Venn diagram was utilized to visualize the intersection of selected targets related to GLE bioactive compounds and EC (Fig. 2A). A total of 83 overlapping targets were acquired as the potential targets for GL treating EC (Table S1). The compound-target network has been established to illustrate the interaction between the bioactive compounds of GL and the potential targets. This network consists of 1 GL node, 26 compound nodes, 83 target nodes, and 165 edges (Fig. 2B). A PPI network comprising 83 nodes and 446 edges was constructed to represent the potential targets. The average degree of the network was 27.9, as shown in Fig. 3A. The hub targets of GL against EC were identified as INS, TP53, AKT1, SRC, MAPK3, MAPK1, ESR1, IL6, HRAS, and CASP3, which had high degrees in the PPI network (Fig. 3B).

### GO and KEGG Analyses

**Fig. 4:**
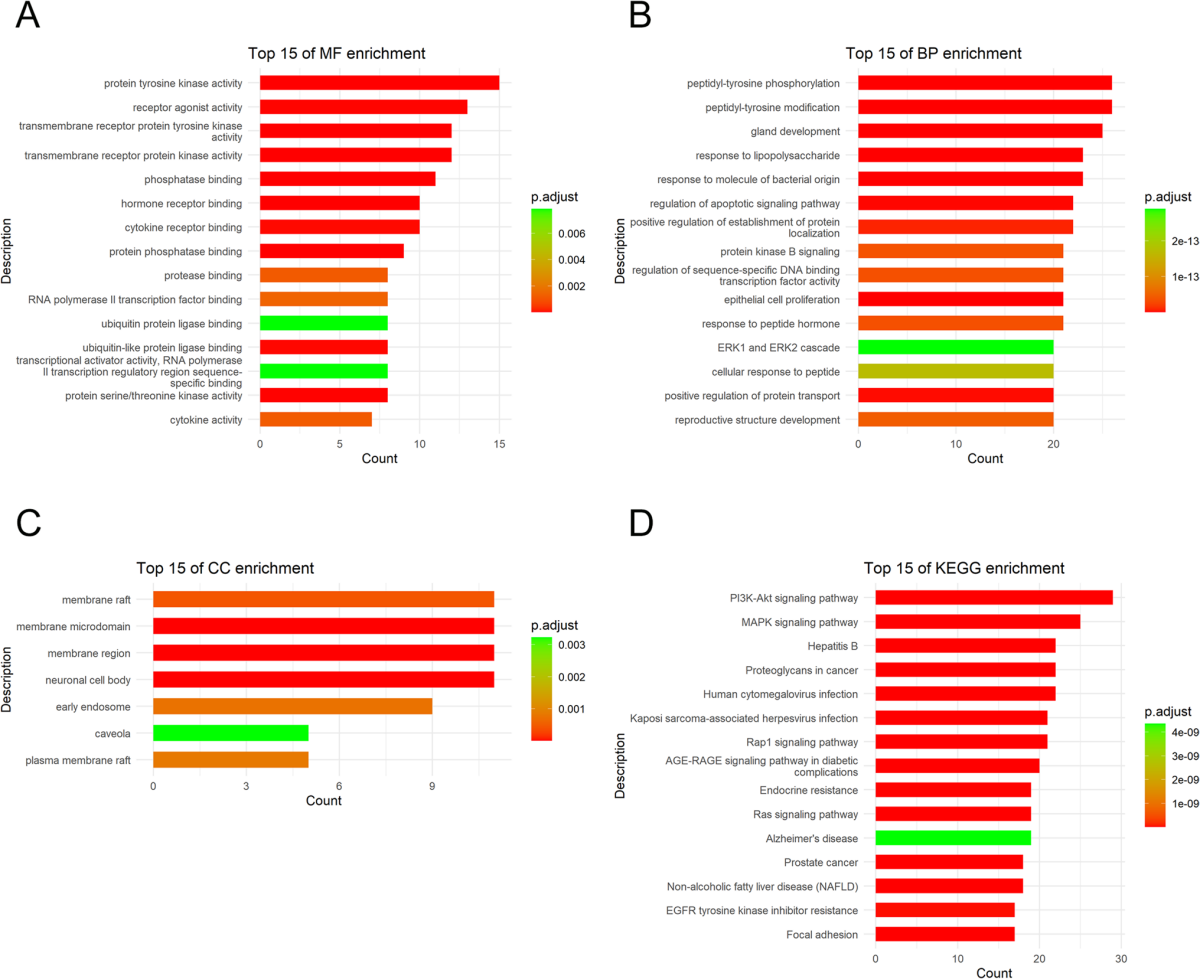
Gene Ontology (GO) and Kyoto Encyclopedia of Genes and Genomes (KEGG) enrichment analyses of 83 potential targets for GL against EC. **A** Top 15 GO-molecular function (MF) terms. **B** Top 15 GO-biological process (BP) terms. **C** Top 15 GO-cellular component (CC) terms. **D** Top 15 KEGG pathways

**Fig. 5:**
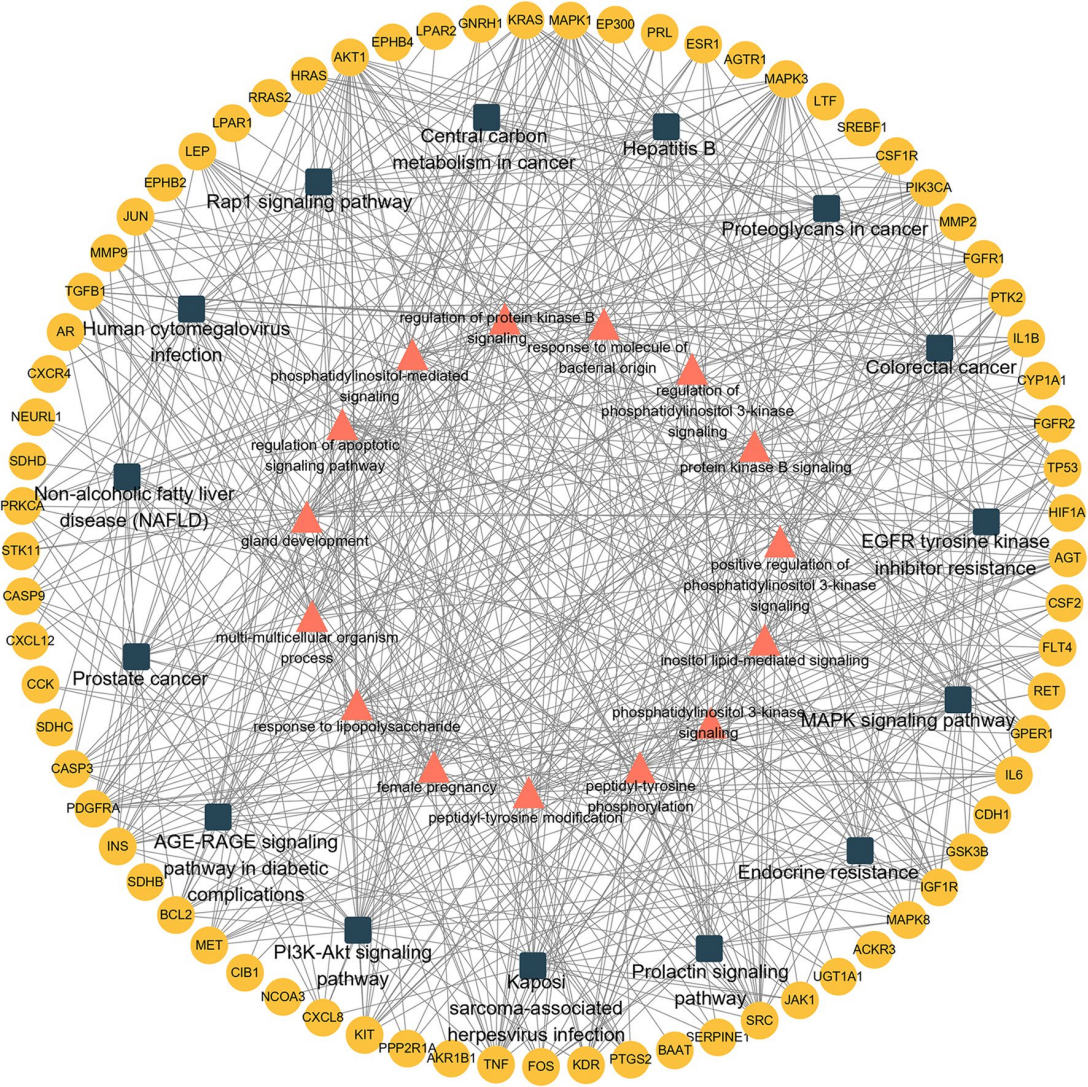
Target-BP-pathway network of GL in the treatment of EC. This network includes 83 potential targets (yellow circles), top 15 GO-BP terms (pink triangles), and top 15 KEGG pathways (dark grey quadrates)

In order to investigate the biological functions and mechanisms underlying the efficacy of GL against EC, 83 potential targets were subjected to GO and KEGG enrichment analyses. Figure 4A illustrates the top 15 GO enrichment terms, highlighting the implication of numerous targets in various molecular functions (MFs) related to protein tyrosine kinase and receptor agonist activity. Biological process (BP) results showed that many targets were involved in peptidyl-tyrosine modification, gland development, and response to lipopolysaccharide (Fig. 4B). Figure 4C depicts the localization pattern of targets in this study, which were primarily found in the membrane microdomain, membrane region, and neuronal cell body according to the cellular component (CC) analysis. Figure 4D was created to illustrate the 15 most significant KEGG pathways in the form of a bar plot diagram, a visual aid complemented by a table of the same pathways (Table S2). The findings revealed that these targets were predominantly associated with PI3K-Akt, MAPK, and Rap1 signaling pathways (Fig. 4D and Table S2). An intricate network (Fig. 5) was constructed, demonstrating the interplay between 83 potential targets, the top 15 BPs, and the top 15 KEGG pathways.

### Pathway Enrichment for Hub Targets

**Fig. 6:**
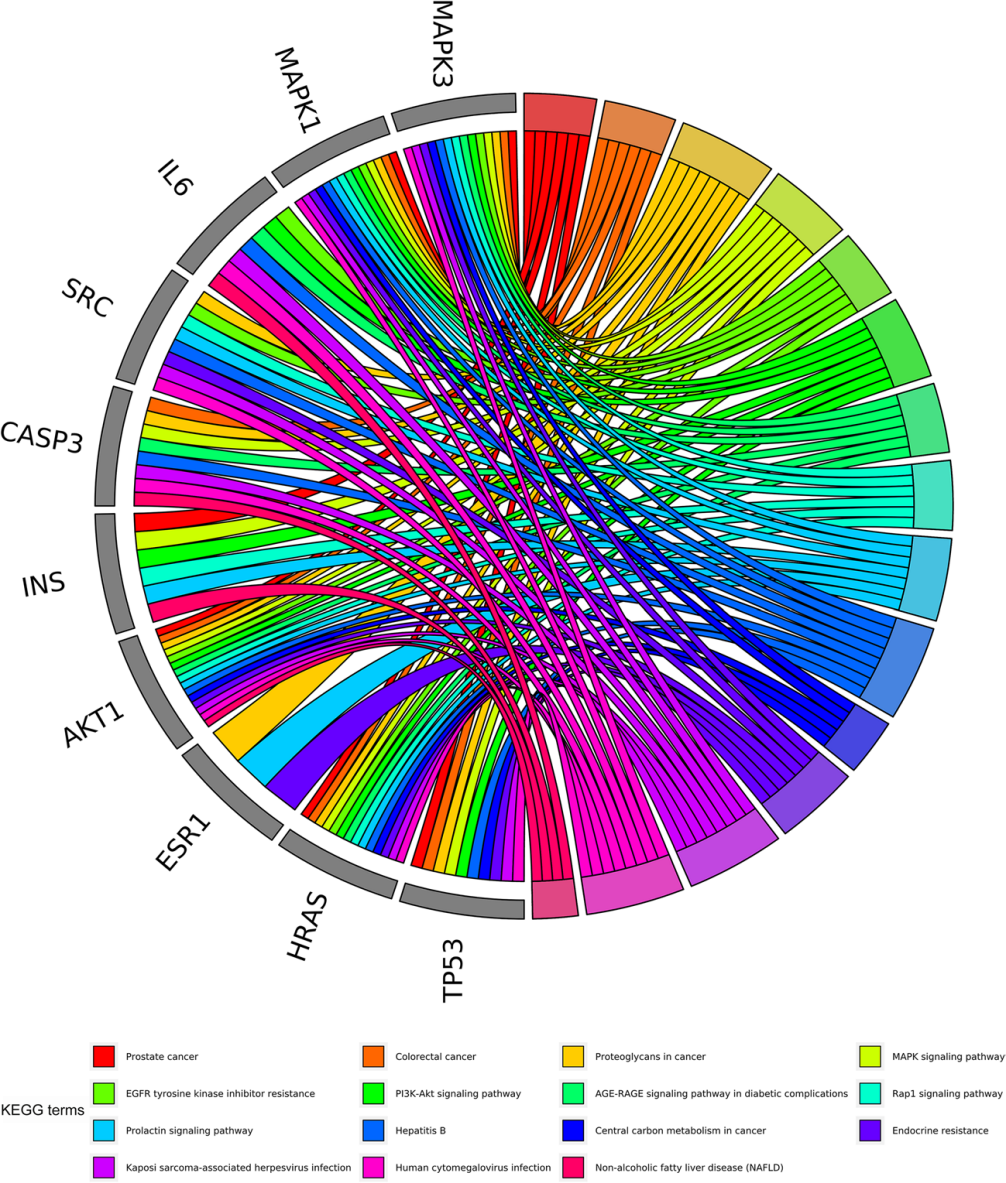
The distribution of top 10 hub targets in top 15 KEGG pathways

**Fig. 7:**
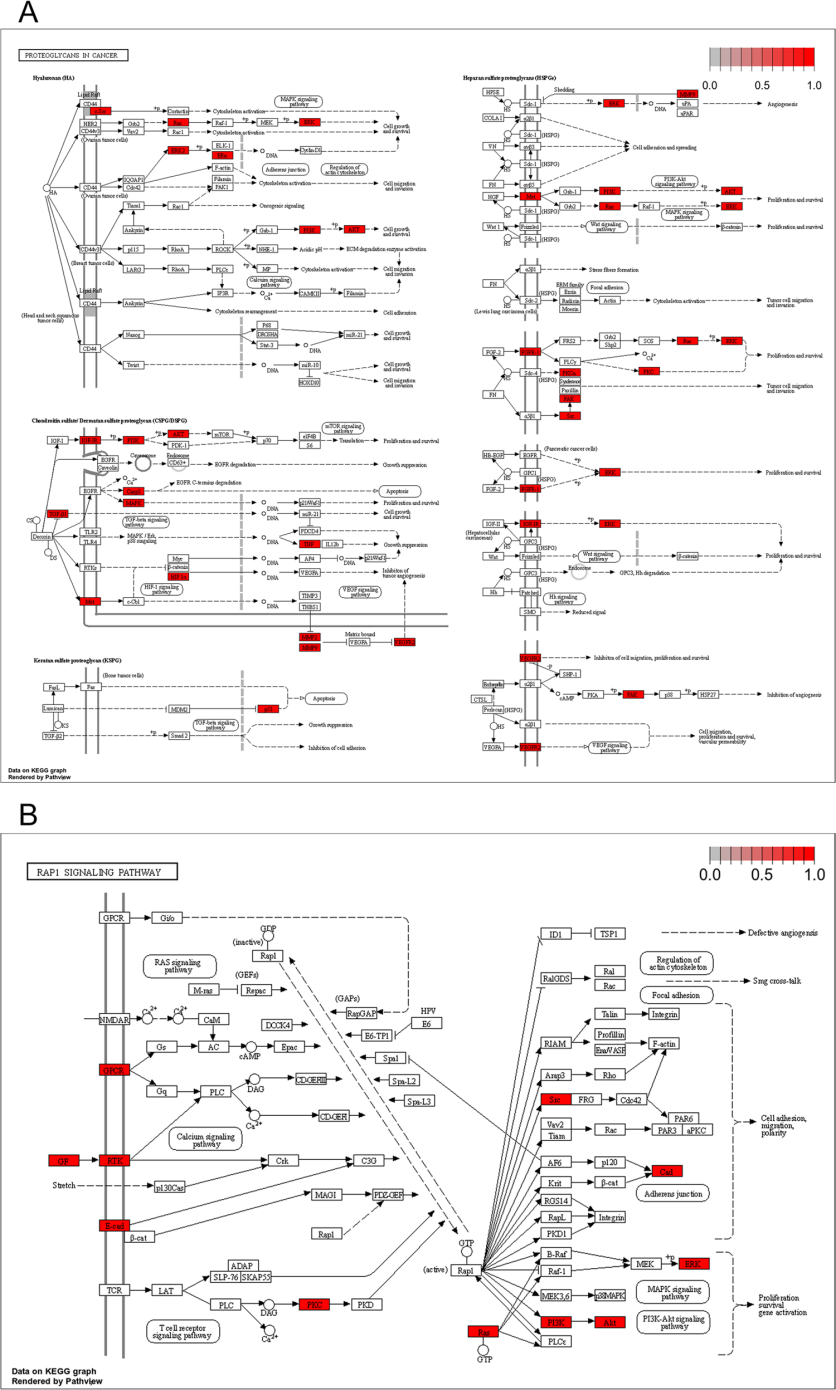
KEGG maps of proteoglycans in cancer (**A**) and Rap1 signaling pathway (**B**)

A chord plot was utilized to visually represent the distribution of the top 10 hub targets in the top 15 KEGG pathways. Figure 6 illustrates that the pathway of proteoglycans in cancer was enriched with eight hub targets, potentially having a significant impact on the treatment of GL on EC. Additionally, six targets were enriched in the Rap1 signaling pathway, which is a crucial player in tumor cell development (Fig. 6). Therefore, the KEGG maps of proteoglycans in cancer and the Rap1 signaling pathway were constructed to confirm the protein interaction and underlying mechanisms of GL against EC. The map of proteoglycans in cancer suggested that this mechanism was involved in multiple targets and pathways, including PI3K-Akt and MAPK pathways, thereby regulating cancer cell proliferation, migration, and survival (Fig. 7A). The map of Rap1 signaling pathway indicated that this pathway was the upstream of PI3K-Akt and MAPK pathways (Fig. 7B).

### GLE Activates the Rap1 Signaling Pathway in EC Cells

**Fig. 8:**
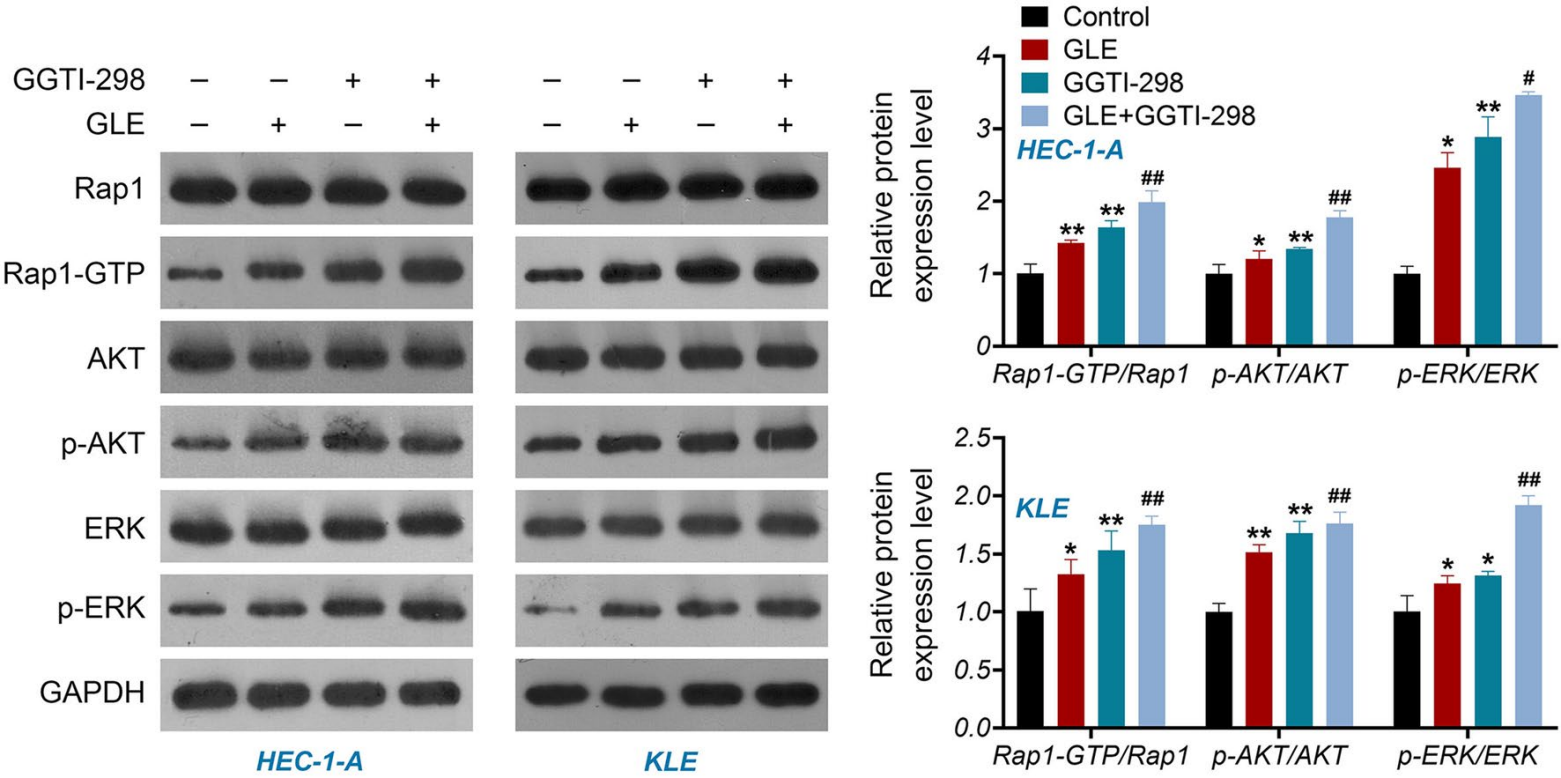
GLE activates the Rap1 signaling pathway in EC cells. The protein expression of Rap1, Rap1-GTP, AKT, p-AKT, ERK2, and p-ERK2 in HEC-1-A and KLE cells was determined by western blotting. HEC-1-A and KLE cells were treated with 1.0 mg/mL GLE and/or 15 μM GGTI-298 for 48 h. Data are presented as the mean ± SD of three independent experiments. *P < 0.05 and **P < 0.01 vs. the control, and ^#^*P* < 0.05 and ^##^*P* < 0.01 vs. the GLE treatment

Based on the network pharmacological analysis, the Rap1 signaling pathway may act as a crucial role in the treatment of GLE on EC. Figure 8 illustrates that the presence of GLE resulted in a significant upregulation in protein expression of Rap1-GTP in HEC-1-A and KLE cells, as opposed to the control samples (*P* < 0.01). p-AKT and p-ERK are both downstream effectors involved in the Rap1 pathway, which were also upregulated by the treatment of GLE (*P* < 0.01). GGTI-298 is a geranylgeranyltransferase I inhibitor preventing the prenylation of Rap1 and increases the level of activated Rap1-GTP. Our research revealed that the utilization of GGTI-298 resulted in a substantial upregulation of Rap1-GTP, p-AKT, and p-ERK2 protein expression in EC cells when compared to the control group (*P* < 0.01). GGTI-298 also potentiated the effect of GLE on activating Rap1 signaling pathway (*P* < 0.05, Fig. 8).

### GLE Suppresses the Malignant Features of EC Cells by Activating Rap1 Signaling Pathway

**Fig. 9:**
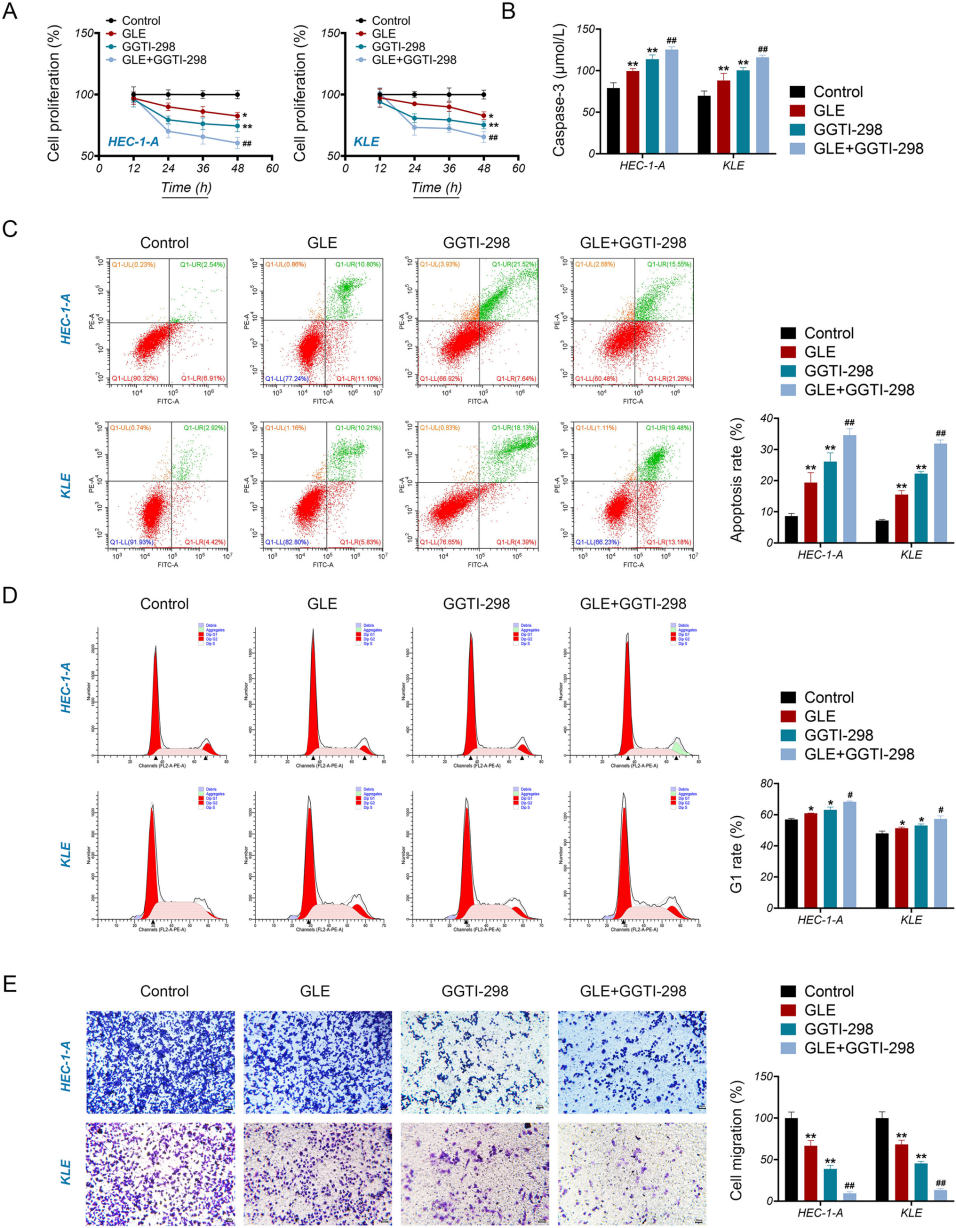
GLE suppresses the malignant progression of EC cells probably via activating the Rap1 signaling pathway. **A** The proliferation of HEC-1-A and KLE cells was measured by CCK-8 assay. HEC-1-A and KLE cells were treated with 1.0 mg/mL GLE and/or 15 μM GGTI-298 for 12, 24, 36, and 48 h. **B** The level of caspase-3 (an apoptosis biomarker) in HEC-1-A and KLE cells was detected by enzyme-linked immunosorbent assay. **C** The apoptosis rate of HEC-1-A and KLE cells was measured by flow cytometry. (**D**) The cell cycle distribution of HEC-1-A and KLE cells was analyzed by flow cytometry. **E** The migration of HEC-1-A and KLE cells was assessed by transwell assay. Scar bar = 50 μm. HEC-1-A and KLE cells were treated with 1.0 mg/mL GLE and/or 15 μM GGTI-298 for 48 h for **B**–**D**. Data are presented as the mean ± SD of three independent experiments. *P < 0.05 and **P <0.01 vs. the control, and ^#^*P* < 0.05 and ^##^*P* < 0.01 vs. the GLE treatment

We further verified whether GLE retards the malignant progression of EC cells by affecting the Rap1 signaling pathway. Our findings indicate that treatment with GLE or GGTI-298 significantly reduced the proliferation ability of HEC-1-A and KLE cells compared to the control cells (*P* < 0.05, Fig. 9A). On the other hand, treatment with GLE or GGTI-298 triggered a significant increase in apoptosis of EC cells. This was manifested through increased levels of caspase-3, which acts as a regulator of apoptosis, as well as a higher rate of programmed cell death, as compared to the control cells (*P* < 0.01, Fig. 9B, C). Treatment with GLE or GGTI-298 led to the arrest of the cell cycle at the G1 phase in EC cells (*P* < 0.01, Fig. 9D). Treatment with GLE or GGTI-298 also resulted in a significant reduction in the migration of EC cells compared to the control group (*P* < 0.05, Fig. 9E). Furthermore, GGTI-298 enhanced the effects of GLE on inhibiting the malignant features of EC cells (*P* < 0.01, Fig. 9A–E).

## Discussion

EC is a prevalent form of cancer in the female reproductive system that has a significant impact on women’s quality of life due to its high morbidity and mortality rates [32]. GL, a medicinal mushroom, owes exceptional antitumor activities towards cancers [33]. Through network pharmacological analysis, it was discovered that the therapeutic targets and molecular mechanism of GL in treating EC are potentially linked to the regulation of the Rap1 signaling pathway. Subsequent in vitro experiments provided further validation that GLE effectively impedes the malignant traits of EC cells. Moreover, the action mechanism of GLE on inhibiting EC progression was involved in the activation of the Rap1 signaling pathway.

Prior studies have indicated that GL demonstrates an antineoplastic impact in various types of cancer by inhibiting the malignant advancement of neoplastic cells [34–36]. GL has been found to possess antitumor properties in glioblastoma cells by suppressing cell proliferation and migration, inducing apoptosis, and causing cell cycle arrest in the S phase [34]. The proliferation of cells is repressed, apoptosis is promoted, and cell cycle is arrested in G2/M phase in liver cancer cells through the action of GLE [36]. Consistent with these findings of GLE on glioblastoma and liver cancer, our research demonstrated that GLE effectively suppresses cell proliferation and migration, while facilitating apoptosis and inducing cell cycle arrest in the G1 phase in EC cell lines. These results indicate that GLE is an effective antitumor drug for EC.

Network pharmacology is a systematic method that is widely applied to interpret potential interaction among disease, bioactive compounds of TCM, and biological mechanisms [37]. In the current study, drugs-active compounds-targets network uncovered that 83 potential targets were closely associated with GL treating EC, in which INS, TP53, AKT1, SRC, MAPK3, MAPK1, ESR1, IL6, HRAS, and CASP3 were top 10 hub targets. It has been reported that TP53, AKT1, SRC, MAPK, ESR1, IL6, and CASP3 are bound up with the pathogenesis of EC and are regarded as therapeutic targets for EC [38–43]. The tumor suppressor TP53 is the most commonly mutated gene in EC [44, 45]. High expression of AKT1, associated with poor prognosis in EC, is reported to regulate progesterone receptor B-dependent transcription and angiogenesis [39]. Increased SRC expression has been observed in advanced EC tissues, contributing to tumor metastasis [38]. The presence of activated MAPK3/1 expression has been detected in normal proliferative phase endometria and EC, suggesting its involvement in actively proliferating endometrial tissues [46]. ESR1, the main mediator of estrogen effects in the endometrium, influences susceptibility and prognosis in EC [47]. Activation of the IL-6 pathway can promote EC cell viability, migration, and invasion [48]. A systematic pan-cancer analysis demonstrated that CASP3 can be a potential target for immunotherapy in various cancers, including EC [49]. Moreover, the GO and KEGG enrichment analyses demonstrated that the hub targets were significantly enriched in proteoglycans in cancer and Rap1 signaling pathways. This provides evidence that these pathways may represent the key mechanisms through which GL exerts its therapeutic effects against EC.

The Rap1 signaling pathway is an essential factor in the mechanisms of tumor growth and metastasis [13]. Rap1 is active when Rap1 binds to GTP (Rap1-GTP), which can be inactivated to Rap1-GDP form by Rap1GAP (a GTPase activating protein) [50]. Several previous studies have reported that increased Rap1-GTP regulated by its negative regulator Rap1GAP can impair the progression of cancers [13, 51, 52]. Activation of Rap1-GTP has been demonstrated to inhibit tumor cell proliferation, metastasis, and angiogenesis in NSCLC [52]. Colorectal cancer cell proliferation, migration, and invasion can be effectively suppressed by inhibiting the expression of Rap1GAP [51]. Our research findings demonstrate that GGTI-298, a GTPase inhibitor, can effectively inhibit cell proliferation and migration, promote apoptosis, and arrest the cell cycle in G1 phase in EC. The results indicate that elevation of Rap1-GTP hinders the malignant development of EC cells. Similar to the effects of GGTI-298, we also found that GLE upregulated the protein level of Rap1-GTP in EC cells, suggesting that GLE can activate the Rap1 signaling pathway in EC cells. Meanwhile, the administration of GGTI-298 augmented the efficacy of GLE in impeding the malignant advancement of EC cells. These findings indicate that GLE may suppress EC progression via activating the Rap1 signaling pathway.

Both AKT and ERK are downstream proteins of the Rap1 signaling pathway. Jin et al. [53] suggested that activation of Rap1 can promote AKT phosphorylation in lung adenocarcinoma. Gao et al. [54] found that the activation of Rap1 is accompanied by increased ERK activity in melanoma. In order to validate the mode of action of GLE in relation to the Rap1 signaling pathway, we assessed the activation of AKT and ERK2 in EC cells. The results demonstrate that GLE upregulates the protein expression of p-AKT and p-ERK2 in EC cells. This result indicates that GLE can activate the AKT/ERK2 following the activation of the Rap1 signaling pathway. Previous studies have been reported that Rap1 can regulate AKT and ERK2 activation, thereby affecting the proliferation and survival of cancer cells [53, 54]. Consequently, our hypothesis is that GLE can impede the malignant advancement of EC by means of stimulating the Rap1 signaling pathway and the subsequent activation of the AKT/ERK2 pathway.

In conclusion, GLE has shown promising potential as a therapeutic option for EC treatment by inhibiting the proliferation and migration of cells as well as inducing apoptosis. Our study has demonstrated that the activation of the Rap1 signaling pathway, validated through experimental verification and network pharmacology, is the underlying mechanism by which GLE exerts its therapeutic effects against EC. These findings offer a promising therapeutic agent for EC treatment and shed light on the underlying mechanisms. However, further confirmation of the antitumor efficacy of GLE on EC requires in vivo experimentation. Additionally, investigating other action mechanisms of GLE against EC, as suggested by network pharmacology, would also be valuable.

### Supplementary Information

Below is the link to the electronic supplementary material.

Supplementary file1 (DOCX 13 KB)

Supplementary file2 (DOCX 12 KB)

## Data Availability

The datasets used and/or analyzed during the current study are available from the corresponding author on reasonable request.

## References

[1] Brooks RA, Fleming GF, Lastra RR, Lee NK, Moroney JW, Son CH, Tatebe K (2019). Current recommendations and recent progress in endometrial cancer. CA Cancer J Clin.

[2] Sung H, Ferlay J, Siegel RL, Laversanne M, Soerjomataram I, Jemal A, Bray F (2021). Global Cancer Statistics 2020: GLOBOCAN estimates of incidence and mortality worldwide for 36 cancers in 185 countries. CA Cancer J Clin.

[3] Tsai YT, Kuo PH, Kuo HP, Hsu CY, Lee YJ, Kuo CL, Liu JY (2021). Ganoderma tsugae suppresses the proliferation of endometrial carcinoma cells via Akt signaling pathway. Environ Toxicol.

[4] Blundell R, Camilleri E, Baral B, Karpiński TM, Neza E, Atrooz OM (2023). The phytochemistry of Ganoderma species and their medicinal potentials. Am J Chin Med.

[5] Abu-Serie MM, Habashy NH, Attia WE (2018). In vitro evaluation of the synergistic antioxidant and anti-inflammatory activities of the combined extracts from Malaysian Ganoderma lucidum and Egyptian Chlorella vulgaris. BMC Complement Altern Med.

[6] Subedi K, Basnet BB, Panday R, Neupane M, Tripathi GR (2021). Optimization of growth conditions and biological activities of Nepalese Ganoderma lucidum strain Philippine. Adv Pharmacol Pharm Sci.

[7] Bai JH, Xu J, Zhao J, Zhang R (2020). Ganoderma lucidum polysaccharide enzymatic hydrolysate suppresses the growth of human colon cancer cells via inducing apoptosis. Cell Transplant.

[8] Barbieri A, Quagliariello V, Del Vecchio V, Falco M, Luciano A, Amruthraj NJ (2017). Anticancer and anti-inflammatory properties of Ganoderma lucidum extract effects on melanoma and triple-negative breast cancer treatment. Nutrients..

[9] Jin H, Song C, Zhao Z, Zhou G (2020). Ganoderma lucidum polysaccharide, an extract from Ganoderma lucidum, exerts suppressive effect on cervical cancer cell malignancy through mitigating epithelial-mesenchymal and JAK/STAT5 signaling pathway. Pharmacology.

[10] Zhang Y (2017). Ganoderma lucidum (Reishi) suppresses proliferation and migration of breast cancer cells via inhibiting Wnt/β-catenin signaling. Biochem Biophys Res Commun.

[11] Hahne JC, Meyer SR, Dietl J, Honig A (2014). The effect of Cordyceps extract and a mixture of Ganoderma lucidum/Agaricus Blazi Murill extract on human endometrial cancer cell lines in vitro. Int J Oncol.

[12] Alemayehu M, Dragan M, Pape C, Siddiqui I, Sacks DB, Di Guglielmo GM, Babwah AV (2013). β-Arrestin2 regulates lysophosphatidic acid-induced human breast tumor cell migration and invasion via Rap1 and IQGAP1. PLoS ONE.

[13] Looi CK, Hii LW, Ngai SC, Leong CO, Mai CW (2020). The role of Ras-associated protein 1 (Rap1) in cancer: bad actor or good player?. Biomedicines..

[14] Xiao L, Lan X, Shi X, Zhao K, Wang D, Wang X, Li F (2017). Cytoplasmic RAP1 mediates cisplatin resistance of non-small cell lung cancer. Cell Death Dis.

[15] Yang Y, Zhang J, Yan Y, Cai H, Li M, Sun K, Wang J (2017). Low expression of Rap1GAP is associated with epithelial-mesenchymal transition (EMT) and poor prognosis in gastric cancer. Oncotarget.

[16] Zhang L, Chenwei L, Mahmood R, van Golen K, Greenson J, Li G, D’Silva NJ (2006). Identification of a putative tumor suppressor gene Rap1GAP in pancreatic cancer. Cancer Res.

[17] Zuo H, Gandhi M, Edreira MM, Hochbaum D, Nimgaonkar VL, Zhang P, Dipaola J (2010). Downregulation of Rap1GAP through epigenetic silencing and loss of heterozygosity promotes invasion and progression of thyroid tumors. Cancer Res.

[18] Wang Y, Zhang Y, Wang Y, Shu X, Lu C, Shao S, Liu X (2021). Using network pharmacology and molecular docking to explore the mechanism of Shan Ci Gu (Cremastra appendiculata) against non-small cell lung cancer. Front Chem.

[19] Wu J, Chen R, Shen H, Yan T, Qian Y, Zhang Y, Huang Z (2021). Transcriptome analysis of ivosidenib-mediated inhibitory functions on non-small cell lung cancer. Front Oncol.

[20] Zhang F, Ni ZJ, Ye L, Zhang YY, Thakur K, Cespedes-Acuña CL, Han J (2021). Asparanin A inhibits cell migration and invasion in human endometrial cancer via Ras/ERK/MAPK pathway. Food Chem Toxicol.

[21] Lee WY, Lee CY, Kim YS, Kim CE (2019). The methodological trends of traditional herbal medicine employing network pharmacology. Biomolecules..

[22] Huang L, Xie D, Yu Y, Liu H, Shi Y, Shi T, Wen C (2018). TCMID 2.0: a comprehensive resource for TCM. Nucleic Acids Res..

[23] Ru J, Li P, Wang J, Zhou W, Li B, Huang C, Li P (2014). TCMSP: a database of systems pharmacology for drug discovery from herbal medicines. J Cheminform.

[24] Ye H, Ye L, Kang H, Zhang D, Tao L, Tang K, Liu X (2011). HIT: linking herbal active ingredients to targets. Nucleic Acids Res..

[25] Bickerton GR, Paolini GV, Besnard J, Muresan S, Hopkins AL (2012). Quantifying the chemical beauty of drugs. Nat Chem.

[26] Liang X, Li H, Li S (2014). A novel network pharmacology approach to analyse traditional herbal formulae: the Liu-Wei-Di-Huang pill as a case study. Mol Biosyst.

[27] Ming Y, Chen J, Xu L, Shi X, Wang X (2018). A novel adaptive ensemble classification framework for ADME prediction. RSC Adv.

[28] Wishart DS, Feunang YD, Guo AC, Lo EJ, Marcu A, Grant JR, Sajed T (2018). DrugBank 5.0: a major update to the DrugBank database for 2018. Nucleic Acids Res..

[29] Li Y, Liu Y, Yang M, Wang Q, Zheng Y, Xu J, Zheng P (2021). A study on the therapeutic efficacy of San Zi Yang Qin decoction for non-alcoholic fatty liver disease and the underlying mechanism based on network pharmacology. Evid Based Complement Alternat Med.

[30] Yang M, Chen J, Xu L, Shi X, Zhou X, An R, Wang X (2018). A network pharmacology approach to uncover the molecular mechanisms of herbal formula Ban-Xia-Xie-Xin-Tang. Evid Based Complement Alternat Med.

[31] Pang Y, Bai G, Zhao J, Wei X, Li R, Li J, Hu S (2022). The BRD4 inhibitor JQ1 suppresses tumor growth by reducing c-Myc expression in endometrial cancer. J Transl Med.

[32] Henley SJ, Ward EM, Scott S, Ma J, Anderson RN, Firth AU, Thomas CC (2020). Annual report to the nation on the status of cancer, part I: national cancer statistics. Cancer.

[33] Ahmad R, Riaz M, Khan A, Aljamea A, Algheryafi M, Sewaket D, Alqathama A (2021). Ganoderma lucidum (Reishi) an edible mushroom; a comprehensive and critical review of its nutritional, cosmeceutical, mycochemical, pharmacological, clinical, and toxicological properties. Phytother Res.

[34] Cheng AY, Chien YC, Lee HC, Hsieh YH, Yu YL (2020). Water-extracted Ganoderma lucidum induces apoptosis and S-phase arrest via cyclin-CDK2 pathway in glioblastoma cells. Molecules..

[35] Wu X, Jiang L, Zhang Z, He Y, Teng Y, Li J, Yuan S (2021). Pancreatic cancer cell apoptosis is induced by a proteoglycan extracted from Ganoderma lucidum. Oncol Lett.

[36] Zhu L, Wu M, Li P, Zhou Y, Zhong J, Zhang Z, Li Y (2020). High-pressure supercritical CO(2) extracts of Ganoderma lucidum fruiting body and their anti-hepatoma effect associated with the Ras/Raf/MEK/ERK signaling pathway. Front Pharmacol.

[37] Tan L, Tu Y, Wang K, Han B, Peng H, He C (2020). Exploring protective effect of Glycine tabacina aqueous extract against nephrotic syndrome by network pharmacology and experimental verification. Chin Med.

[38] Hu Y, Wu AY, Xu C, Song KQ, Wang WJ, Yin X, Di W (2019). MicroRNA-449a inhibits tumor metastasis through AKT/ERK1/2 inactivation by targeting steroid receptor coactivator (SRC) in endometrial cancer. J Cancer.

[39] Huo X, Sun H, Liu Q, Ma X, Peng P, Yu M, Zhang Y (2019). Clinical and expression significance of AKT1 by co-expression network analysis in endometrial cancer. Front Oncol.

[40] Karaca B, Bakır E, Yerer MB, Cumaoğlu A, Hamurcu Z, Eken A (2021). Doxazosin and erlotinib have anticancer effects in the endometrial cancer cell and important roles in ERα and Wnt/β-catenin signaling pathways. J Biochem Mol Toxicol.

[41] Li X, Li H, Pei X, Zhou Y, Wei Z (2021). CCDC68 upregulation by IL-6 promotes endometrial carcinoma progression. J Interferon Cytokine Res.

[42] Reske JJ, Wilson MR, Holladay J, Siwicki RA, Skalski H, Harkins S, Adams M (2021). Co-existing TP53 and ARID1A mutations promote aggressive endometrial tumorigenesis. PLoS Genet.

[43] Xiao H, Zhang Z, Peng D, Wei C, Ma B (2021). Type II transmembrane serine proteases 4 (TMPRSS4) promotes proliferation, invasion and epithelial-mesenchymal transition in endometrial carcinoma cells (HEC1A and Ishikawa) via activation of MAPK and AKT. Anim Cells Syst (Seoul).

[44] Kandoth C, Schultz N, Cherniack AD, Akbani R, Liu Y, Shen H, Robertson AG (2013). Integrated genomic characterization of endometrial carcinoma. Nature.

[45] Leslie KK, Filiaci VL, Mallen AR, Thiel KW, Devor EJ, Moxley K, Richardson D (2021). Mutated p53 portends improvement in outcomes when bevacizumab is combined with chemotherapy in advanced/recurrent endometrial cancer: an NRG Oncology study. Gynecol Oncol.

[46] Kashima H, Shiozawa T, Miyamoto T, Suzuki A, Uchikawa J, Kurai M, Konishi I (2009). Autocrine stimulation of IGF1 in estrogen-induced growth of endometrial carcinoma cells: involvement of the mitogen-activated protein kinase pathway followed by up-regulation of cyclin D1 and cyclin E. Endocr Relat Cancer.

[47] Blanchard Z, Vahrenkamp JM, Berrett KC, Arnesen S, Gertz J (2019). Estrogen-independent molecular actions of mutant estrogen receptor 1 in endometrial cancer. Genome Res.

[48] Che Q, Xiao X, Xu J, Liu M, Lu Y, Liu S, Dong X (2019). 17β-Estradiol promotes endometrial cancer proliferation and invasion through IL-6 pathway. Endocr Connect.

[49] Zhou Z, Xu S, Jiang L, Tan Z, Wang J (2022). A systematic pan-cancer analysis of CASP3 as a potential target for immunotherapy. Front Mol Biosci.

[50] Jaśkiewicz A, Pająk B, Orzechowski A (2018). The Many Faces of Rap1 GTPase. Int J Mol Sci..

[51] Li H, Liang J, Wang J, Han J, Li S, Huang K, Liu C (2021). Mex3a promotes oncogenesis through the RAP1/MAPK signaling pathway in colorectal cancer and is inhibited by hsa-miR-6887-3p. Cancer Commun (Lond).

[52] Zhao Z, Liu B, Sun J, Lu L, Liu L, Qiu J, Li Q (2019). Scutellaria flavonoids effectively inhibit the malignant phenotypes of non-small cell lung cancer in an Id1-dependent manner. Int J Biol Sci.

[53] Jin X, Di X, Wang R, Ma H, Tian C, Zhao M, Cong S (2019). RBM10 inhibits cell proliferation of lung adenocarcinoma via RAP1/AKT/CREB signalling pathway. J Cell Mol Med.

[54] Gao L, Feng Y, Bowers R, Becker-Hapak M, Gardner J, Council L, Linette G (2006). Ras-associated protein-1 regulates extracellular signal-regulated kinase activation and migration in melanoma cells: two processes important to melanoma tumorigenesis and metastasis. Cancer Res.

