# Supplementary material for: The Efficacy of Ganoderma lucidum Extracts on Treating Endometrial Cancer: A Network Pharmacology Approach
Source: Reprod Sci. 2024 Mar 6;31(7):1881–94. doi: 10.1007/s43032-024-01500-3 (PMC11217070; doi:10.1007/s43032-024-01500-3)
Supplement: Supplementary file 1 — Supplementary file1 (DOCX 13 KB) [file 43032_2024_1500_MOESM1_ESM.docx]

**Table S1.** 83 potential targets of GL against EC.

| ID | Name | ID | Name | ID | Name | ID | Name |
| --- | --- | --- | --- | --- | --- | --- | --- |
| 57007 | ACKR3 | 1543 | CYP1A1 | 3716 | JAK1 | 5468 | PPARG |
| 183 | AGT | 2033 | EP300 | 3725 | JUN | 5518 | PPP2R1A |
| 185 | AGTR1 | 2048 | EPHB2 | 3791 | KDR | 5578 | PRKCA |
| 231 | AKR1B1 | 2050 | EPHB4 | 3815 | KIT | 5617 | PRL |
| 207 | AKT1 | 2099 | ESR1 | 3845 | KRAS | 5743 | PTGS2 |
| 367 | AR | 2260 | FGFR1 | 3952 | LEP | 5747 | PTK2 |
| 570 | BAAT | 2263 | FGFR2 | 1902 | LPAR1 | 5979 | RET |
| 596 | BCL2 | 2271 | FH | 9170 | LPAR2 | 22800 | RRAS2 |
| 794 | CALB2 | 2324 | FLT4 | 4057 | LTF | 6390 | SDHB |
| 836 | CASP3 | 2353 | FOS | 5594 | MAPK1 | 6391 | SDHC |
| 842 | CASP9 | 50943 | FOXP3 | 5595 | MAPK3 | 6392 | SDHD |
| 885 | CCK | 2796 | GNRH1 | 5599 | MAPK8 | 5054 | SERPINE1 |
| 999 | CDH1 | 2798 | GNRHR | 4233 | MET | 6714 | SRC |
| 1080 | CFTR | 2852 | GPER1 | 4311 | MME | 6720 | SREBF1 |
| 10519 | CIB1 | 2932 | GSK3B | 4313 | MMP2 | 6794 | STK11 |
| 1436 | CSF1R | 3091 | HIF1A | 4318 | MMP9 | 7040 | TGFB1 |
| 1437 | CSF2 | 3265 | HRAS | 8202 | NCOA3 | 7124 | TNF |
| 6387 | CXCL12 | 3480 | IGF1R | 9148 | NEURL1 | 7157 | TP53 |
| 3576 | CXCL8 | 3553 | IL1B | 5047 | PAEP | 54658 | UGT1A1 |
| 7852 | CXCR4 | 3569 | IL6 | 5156 | PDGFRA | 22882 | ZHX2 |
| 1583 | CYP11A1 | 3630 | INS | 5290 | PIK3CA |  |  |

**Abbreviations:** GL, *Ganoderma lucidum*; EC, endometrial cancer.
