# Supplementary material for: The Efficacy of Ganoderma lucidum Extracts on Treating Endometrial Cancer: A Network Pharmacology Approach
Source: Reprod Sci. 2024 Mar 6;31(7):1881–94. doi: 10.1007/s43032-024-01500-3 (PMC11217070; doi:10.1007/s43032-024-01500-3)
Supplement: Supplementary file 2 — Supplementary file2 (DOCX 12 KB) [file 43032_2024_1500_MOESM2_ESM.docx]

**Table S2.** Top 15 KEGG pathways of GL against EC.

| ID | Description | p.adjust | Count |
| --- | --- | --- | --- |
| hsa04933 | AGE-RAGE signaling pathway in diabetic complications | 1.87E-19 | 20 |
| hsa01522 | Endocrine resistance | 1.97E-18 | 19 |
| hsa04151 | PI3K-Akt signaling pathway | 1.97E-18 | 29 |
| hsa05161 | Hepatitis B | 2.38E-18 | 22 |
| hsa01521 | EGFR tyrosine kinase inhibitor resistance | 1.57E-17 | 17 |
| hsa05215 | Prostate cancer | 1.83E-17 | 18 |
| hsa05205 | Proteoglycans in cancer | 2.35E-16 | 22 |
| hsa04010 | MAPK signaling pathway | 2.35E-16 | 25 |
| hsa05167 | Kaposi sarcoma-associated herpesvirus infection | 9.33E-16 | 21 |
| hsa05163 | Human cytomegalovirus infection | 1.33E-15 | 22 |
| hsa04015 | Rap1 signaling pathway | 4.40E-15 | 21 |
| hsa05210 | Colorectal cancer | 2.77E-14 | 15 |
| hsa04932 | Non-alcoholic fatty liver disease (NAFLD) | 2.77E-14 | 18 |
| hsa05230 | Central carbon metabolism in cancer | 3.06E-14 | 14 |
| hsa04917 | Prolactin signaling pathway | 8.73E-13 | 13 |

**Abbreviations:** KEGG, Kyoto Encyclopedia of Genes and Genomes ; GL, *Ganoderma lucidum*; EC, endometrial cancer.
